# Efficacy of Transcatheter Renal Arterial Embolization to Contract Renal Size and Increase Muscle Mass in Patients with Polycystic Kidney Disease

**DOI:** 10.3390/diagnostics16020302

**Published:** 2026-01-17

**Authors:** Che-Ming Lin, Tai-Shuan Lai, Ting-Wei Liao, Trianingsih, Ying-Hui Wu, Chun-Jung Cheng, Chih-Horng Wu

**Affiliations:** 1 Department of Radiology, Shuang Ho Hospital, Taipei Medical University, New Taipei City 235, Taiwan; 16610@s.tmu.edu.tw; 2 Department of Radiology, School of Medicine, College of Medicine, Taipei Medical University, Taipei 110301, Taiwan; 3 Departments of Internal Medicine, National Taiwan University Hospital, Taipei 100225, Taiwan; d99849016@ntu.edu.tw; 4 Department of Medical Imaging, National Taiwan University Hospital, Taipei 100225, Taiwan; twliao1219@gmail.com (T.-W.L.); zorawu@ntuh.gov.tw (Y.-H.W.); cjcheng@ntuh.gov.tw (C.-J.C.); 5 Department of Radiology, Faculty of Medicine, Public Health and Nursing, Universitas Gadjah Mada, Dr. Sardjito General Hospital, Yogyakarta 55284, Indonesia; triayunan@gmail.com; 6 Center of Minimal-Invasive Interventional Radiology, National Taiwan University Hospital, Taipei 100225, Taiwan; 7 Hepatitis Research Center, National Taiwan University Hospital, Taipei 100225, Taiwan

**Keywords:** transarterial embolization, muscle mass, sarcopenia, dialysis, polycystic kidney disease

## Abstract

**Background/Objectives**: Autosomal dominant polycystic kidney disease (ADPKD) is a major cause of end-stage kidney disease (ESKD), accounting for approximately 5–10% of patients receiving dialysis worldwide. The large and numerous cysts in the liver and kidneys cause abdominal distention and poor appetite. Previous studies showed that renal arterial embolization (RAE) reduces total kidney volume (TKV), increases appetite, and improves quality of life. This article aims to evaluate the efficacy of RAE in increasing psoas muscle (PM) and paraspinal muscle (PS) mass in patients with polycystic kidney disease. **Methods**: A retrospective study was conducted from May 2016 to December 2020. Thirty-five patients with PKD and ESKD who received RAE were enrolled. The clinical data, including age, sex, body weight, abdominal circumference, and laboratory results, including albumin, creatinine, estimated glomerular filtration rate, and dialysis vintage, were collected. TKV was calculated with the ellipsoid formula method, and muscle mass was measured with bilateral PM and PS areas at the third lumbar level. The associated clinical, laboratory, and imaging data were compared before and after RAE. **Results**: There were 19 females and 16 males with a mean age of 59.9 for the final analysis. There were significant changes between baseline and 3-month, 6-month, 12-month after RAE, such as a decrease in TKV (4684 ± 3361 vs. 4079 ± 3456, 3675 ± 3401, 2459 ± 1706 mL, all *p* < 0.001), an increase in the PM area (12.6 ± 5.8 vs. 13.3 ± 5.7, 14.7 ± 6.9, 14.3 ± 7.1 cm^2^, all *p* < 0.05), but no difference in body weight, body mass index, albumin, hemoglobin, creatinine, or estimated glomerular filtration rate. The increase in the PM and PS was more obvious in the sarcopenic group than in the non-sarcopenic group in the 12-month follow-up (*p* = 0.001 and 0.016 vs. *p* = 0.205 and 0.259). **Conclusions**: RAE effectively reduces TKV, increases PM and PS mass, and serves as a candidate to reverse muscle loss in patients with PKD.

## 1. Introduction

Patients with chronic kidney disease (CKD) and those who undergo dialysis usually present with sarcopenia [1]. A study reported that 20% of patients with end-stage kidney disease (ESKD) undergoing long-term hemodialysis experienced muscle mass loss [2], and another study indicated that patients with muscle wasting had poorer quality of life and prognosis than those without muscle mass loss [3]. In addition, recent studies have demonstrated that CKD is a chronic catabolic condition caused by a persistent imbalance between protein degradation and synthesis in skeletal muscle [4] and is associated with various inflammatory biomarkers [5], a condition known as malnutrition–inflammation (MIA) syndrome. Therefore, exercise, nutrition supplements, and anti-inflammation therapies may help attenuate muscle loss in patients with CKD. In addition, optimization of dialysis therapy, including achieving dialysis adequacy targets such as Kt/V and the use of biocompatible hemodialysis membranes, may further contribute to improving nutritional status and reducing inflammation in patients with CKD [6,7,8].

Polycystic kidney disease (PKD) is the most common hereditary disease causing CKD [9]. Large and numerous cysts in the liver and kidneys cause abdominal distention and poor appetite, accelerating muscle mass loss. In addition, organomegaly also limits physical activity and worsens poor muscle strength and performance. A recent study has demonstrated the inverse correlation between abdominal muscle mass and total kidney volume (TKV) [10]. Therefore, it is challenging to improve sarcopenia and increase muscle mass in patients with PKD.

Ubara et al. first reported a case of PKD with long-term hemodialysis receiving renal artery embolization (RAE) and a series demonstrating RAE reduced the size of numerous cysts in the kidneys and a 46% to 54% decrease in TKV [11,12]. In addition, Yamakoshi et al. showed that RAE could improve lung function in patients with PKD. Suwabe et al. demonstrated that RAE to PKD effectively improved the quality of life and alleviated the symptoms of abdominal fullness and poor appetite [13]. Petitpierre and Del Tatoo et al. also found that some PKD patients contraindicated for kidney transplantation because of excessive renal volume had successful decreases in TKV, making their contraindications withdrawn in 5.6 to 19.5 months on average after RAE [14,15]. However, Ubara et al. and another recent study by Sakuhara have reported that dry weight in patients with PKD continuously decreased in the first half-year and gradually increased six months after RAE [12,16]. These results do not agree with the finding of TKV reduction, and the inconsistency might be explained by changes in body composition, such as muscle gain, which may be masked by the measurement of dry weight only.

Therefore, we hypothesize that RAE can decrease TKV and increase abdominal muscle mass. However, no previous study has quantified the muscle mass change before and after RAE in patients with PKD through cross-sectional imaging. This study aimed to measure the psoas muscle (PM) and paraspinal muscle (PS) areas at the third lumbar vertebral (L3) level at baseline, 3-month, 6-month, and 12-month images after RAE with adjustment of body height (BH) and to investigate the correlation between TKV and muscle mass change.

## 2. Materials and Methods

### 2.1. Study Population

**Figure 1:**
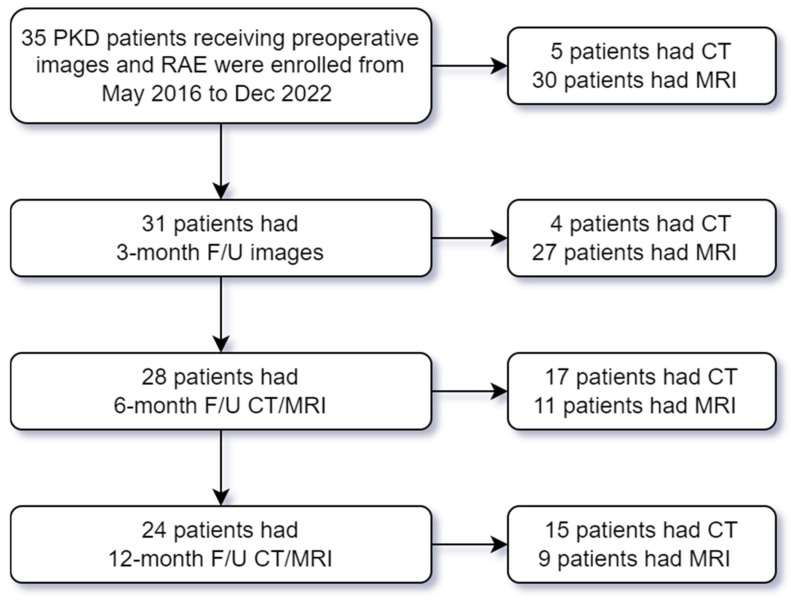
Flowchart of patient enrollment. PKD: polycystic kidney disease; RAE: renal artery embolization; CT: computed tomography; MRI: magnetic resonance imaging; and F/U: follow-up.

A single-center retrospective cohort study was conducted from May 2016 to December 2022 at the National Taiwan University Hospital (NTUH). We enrolled adult patients with PKD under hemodialysis or peritoneal dialysis. The diagnosis of PKD was based on Ravine’s criteria [17]. The inclusion criteria were as follows: (1) patients who underwent computed tomography (CT) or magnetic resonance imaging (MRI) for pre-procedural evaluation, and (2) patients who received RAE within two weeks after imaging evaluation. Follow-up CT or MRI examinations were performed at 3, 6, and 12 months after RAE, and patients were followed until December 2023. Clinical characteristics, laboratory data, and CT or MRI images were retrospectively collected for analysis. The exclusion criteria included the following: (1) incomplete clinical or laboratory data, (2) missing or inadequate CT or MRI images for body composition analysis, and (3) loss to follow-up. The flowchart of patient enrollment is shown in Figure 1. Informed consent was obtained from all subjects involved in the study. This study was approved by the Institutional Review Board of NTUH (No. 201512117RINB and No. 202006139RINA) and registered at ClinicalTrial.gov (NCT05215964).

### 2.2. Study Variables and Imaging Acquisition

Clinical data, including age, sex, body weight (BW), BH, body mass index (BMI), abdominal circumference (AC), and laboratory data (albumin, hemoglobin, and creatinine levels and estimated glomerular filtration rate [eGFR]) were collected from electronic medical records within two weeks of CT or MRI scans.

Primary outcomes of this study were the changes in TKV and skeletal muscle mass indices after RAE, including PM and PS area and index measured on CT or MRI. Secondary outcomes included changes in anthropometric parameters (BW, BMI, and AC) and laboratory parameters (serum albumin, hemoglobin, and creatinine levels) during follow-up.

If patients had no contraindication for MRI (e.g., claustrophobia, metal implants, or pacemakers) or could tolerate multiple sessions of suspended respiration for image acquisition, we performed MRI because of the absence of radiation exposure during the examination. A 3.0-Tesla unit (Magnetom Verio; Siemens Medical Solutions, Erlangen, Germany) was used for MRI. We performed the following sequences: chemical-shift imaging (Dixon method with the following parameters: repetition time (TR): 9.65 ms/time to echo (TE): 2.45 ms for IP and 3.67 ms for OP, 5 mm slice thickness), coronal T2 half-Fourier acquisition single-shot turbo spin-echo (HASTE) with a TR of 2000 ms, TE of 92 ms, and a slice thickness of 3 mm for the abdomen and pelvis to observe the cyst extension and to determine TKV [18]. In addition, T1 and T2-weighted axial images with fat suppression and a slice thickness of 5 mm were also collected from the xiphoid process to pubic symphysis.

CT scan was performed only in patients with contraindications for MRI or who could not suspend respiration. A 64-slice CT scanner (Lightspeed VCT; GE Medical System, Waukesha, WI, USA) was used with the following parameters: 120 Kv, dose modulation according to body size, and reconstruction with a 1 mm slice thickness. Then, the coronal and axial images of the abdomen and pelvis with 3 mm and 5 mm thickness, respectively. CT or MRI was used to evaluate TKV, PM, and PS muscle, depending on patient compliance and imaging availability. Muscle and kidney measurements were performed using modality-specific, previously reported method [10]. CT- and MRI-based measurements were analyzed separately in the serial follow-up of the same patient, and no direct comparison between the two imaging modalities was intended. We performed only CT or MRI images without contrast injection to avoid contrast-induced nephropathy, which refers to acute deterioration of renal function after iodine-based contrast exposure for CT, or nephrogenic systemic fibrosis, a rare but serious systemic fibrotic disorder associated with gadolinium-based contrast agents for MRI in patients with advanced kidney disease.

### 2.3. Imaging Processing

**Figure 2:**
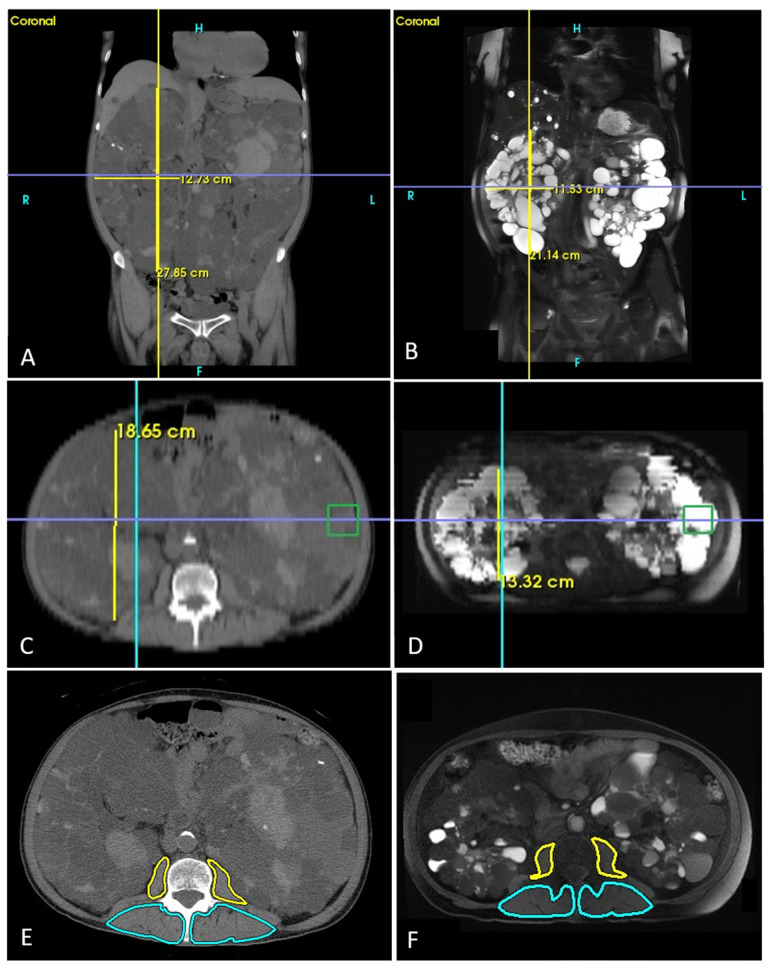
TKV was estimated, and the PM (yellow contour)/PS (blue contour) areas were measured in the same patient of pre-operative CT (**A**,**C**,**E**), 12-month follow-up MRI (**B**,**D**,**F**). TKV: total kidney volume; PM: psoas muscle; PS: paraspinal muscle; CT: computed tomography; and MRI: magnetic resonance imaging.

TKV was estimated with an ellipsoid formula through multiplanar reformation and measured using the SoliPACS Web Viewer (EBM Technologies, Taipei, Taiwan). First, the maximal longitudinal length (L) of the kidney was determined on coronal CT or T2 HASTE with tilted coronal slices parallel to the long renal axis (Figure 2A,B). Next, the maximal width (W) was determined perpendicular to the L in the same plane where the L was localized. Finally, the maximal depth (D) was determined perpendicular to the L in a sagittal thick slice (Figure 2C,D) [18]. As a result, TKV was estimated as follows:

(1) $$Estimated\;TKV\left(mL\right)=\frac{\pi }{6}\times L\left(cm\right)\times W\left(cm\right)\times D\left(cm\right)$$

Non-enhanced CT images and T1-weighted axial images with fat suppression MRI at the L3 level were analyzed to determine PM and PS areas, also processed by SoliPACS Web Viewer (EBM Technologies, Taipei, Taiwan). Contours were obtained using a manual tracing method (Figure 2E,F). A radiologist (C.-H.W.) with 10 years of experience in abdominal imaging processed the images based on the method described previously [19]. The PM and PS indices were calculated as follows:

(2) PM and PS indices(cm2m2)=PM and PS areas(cm2)BH(m2)

If patients had a PM index < 6.36 cm^2^/m^2^ in males or <3.92 in females, they were diagnosed with sarcopenia based on a previous study in the Asian population [20].

### 2.4. Renal Artery Embolization (RAE)

RAE was performed for PKD patients with symptoms such as abdominal distention or hematuria. RAE is a minimally invasive procedure in patients under hemodialysis or peritoneal dialysis in our institute [21]. The RAE was performed via transfemoral puncture after groin area shaving, disinfection with iodine, and local anesthesia with lidocaine. A guidewire is inserted through the femoral artery to the abdominal aorta, and a 5-French sheath (Radiofocus Introducer II; Terumo Cooperation, Tokyo, Japan) is placed at the femoral artery. The renal arteries were catheterized with a 5-French reverse-curve angiographic catheter (RLG, Cook Beacon Tip 5.0 Fr Angiographic Catheter; COOK Medical, Bloomington, IN, USA). While leaving the reverse-curve angiographic catheter placed at either one of the renal arteries, a 2.7-French microcatheter kit (Progreat; Terumo Cooperation, Tokyo, Japan) was inserted into the branches of the renal arteries, which are embolized with multiple platinum pushable coils (Nester and Tornado 0.018″ Microcoil; COOK Medical, Bloomington, IN, USA) and detachable coils (Interlock-18; Boston Scientific, Marlborough, MA, USA or Concerto detachable coil systems; Medtronic, Minneapolis, MN, USA) from distal to stump of renal arteries. We usually left 0.5 to 1.0 cm renal arteries stump without embolization for possible surgical intervention in the future. After embolization, the sheath was removed, and the puncture site of the femoral artery was compressed for at least 5 min, followed by sandbag compression for 4 h and bedrest for 8 h. No major procedure-related complications were observed. Mild post-procedural pain was reported in some patients and was adequately controlled with analgesics. No clinically significant anemia or procedure-related bleeding requiring blood transfusion was noted.

### 2.5. Statistical Analysis

Data were analyzed using the following tools: Excel 2016 (Microsoft, Redmond, WA, USA) and R 3.4.3. Categorical and continuous variables were compared using the chi-square test and Student’s *t*-test, respectively. Continuous data were expressed as mean ± standard deviation. Before performing paired *t*-tests, the normality of continuous variables was assessed using the Shapiro–Wilk test. A paired *t*-test was used to compare pre- and post-procedural measurements. A two-sided *p* value < 0.05 was considered statistically significant. We determined the required sample size to be approximately 8 for continuous variables with a paired *t*-test, assuming a significance of 5% (α = 0.05), a power of 95% (β = 0.05), a mean of 1500 mL with a standard deviation of 1000 mL for TKV. A *p*-value of <0.05 was considered statistically significant.

## 3. Results

### 3.1. Clinical and Laboratory Assessment

We consecutively recruited 35 patients (19 females and 16 males) with a mean age of 59.9 (41–84) years. No patients had mortality during follow-up, and the mean follow-up period was 23.3 months. Among these patients, the indications for renal artery embolization included abdominal distension (*n* = 29) and hematuria (*n* = 6). Based on symptom severity and laterality, 22 patients underwent bilateral RAE, 7 underwent right-sided RAE, and 6 underwent left-sided RAE. Twenty-eight patients received hemodialysis, and 7 patients had peritoneal dialysis. The sarcopenic group has a significantly larger age, lower BMI, PM area/index, and creatinine level. The peritoneal dialysis group only has a significant higher creatinine level. The demographic characteristics of the patients are presented in Table 1.

### 3.2. TKV and Muscle Mass Change After RAE

**Figure 3:**
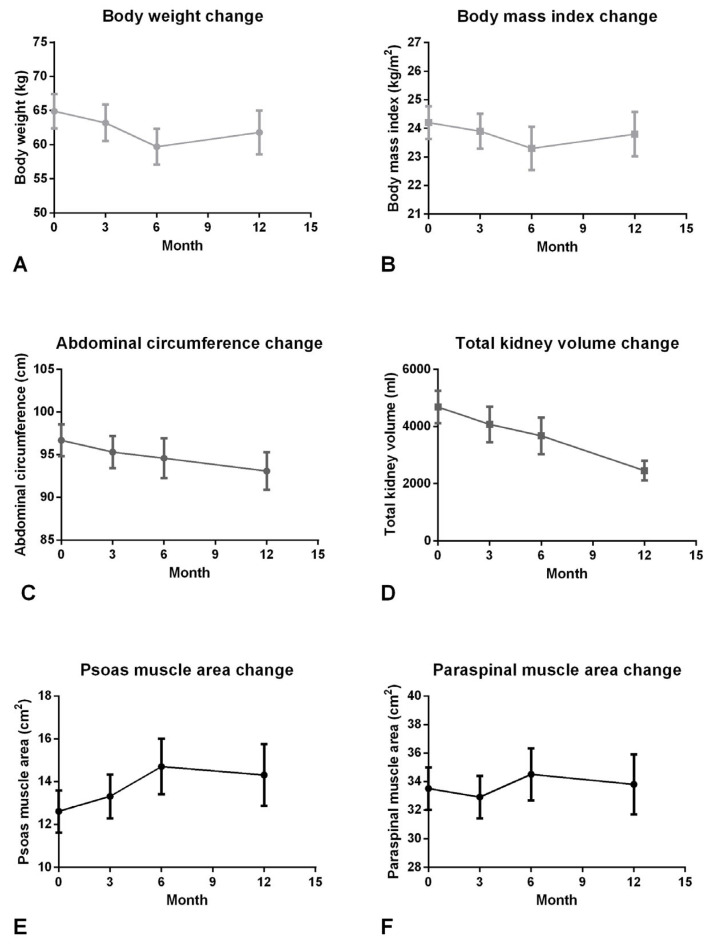
Changes in body weight, body mass index, abdominal circumference, total kidney volume, psoas, and paraspinal muscle areas. Body weight and body mass index showed a decrease during the first six months, followed by a regain at 12 months (**A**,**B**). Abdominal circumference and total kidney volume continued to decrease throughout the 12-month follow-up period (**C**,**D**). Psoas and paraspinal muscle areas reached their peak at the 6-month time point (**E**,**F**).

TKV decreased significantly in 3-, 6-, and 12-month follow-up after RAE compared to baseline images (4684 ± 3361 vs. 4079 ± 3456, 3675 ± 3401, 2459 ± 1706, all *p* < 0.001; Table 2). The trend of TKV decreases continuously from baseline to 12-month follow-up (Figure 3D). During the follow-up period, no patients required subsequent traditional surgical intervention after RAE. One patient underwent renal transplantation during follow-up. Notably, imaging demonstrated an increased available space in the iliac fossa for graft kidney placement following RAE. Furthermore, the PM area in 3, 6, and 12 months after RAE was significantly larger than the baseline (12.6 ± 5.8 vs. 13.3 ± 5.7, 14.7 ± 6.9, 14.3 ± 7.1, all *p* < 0.05; Table 2). The PM area continuously increased from baseline to 3-month and 6-month follow-up and became stable after 6-month to 12-month follow-up (Figure 3E). Similarly, the PS area significantly increased from baseline to 6-month follow-up (33.5 ± 8.8 vs. 34.5 ± 9.7, *p* = 0.013; Figure 3F). On the other hand, there was non-significant decrease in BW and BMI at the 6-month follow-up after RAE (64.9 ± 14.8 vs. 59.7 ± 13.9, *p* = 0.178 and 24.2 ± 3.4 vs. 23.3 ± 4.0, *p* = 0.069; Figure 3A,B; Table 2), and there was also a non-significantly continuous decrease trend in AC (96.7 ± 11.0 vs. 95.3 ± 10.4, *p* = 0.147; 94.6 ± 12.3, *p* = 0.095; 93.1 ± 10.8, *p* = 0.380; Figure 3C). The albumin, hemoglobin, creatinine, and eGFR had no significant change before and after the procedure.

### 3.3. Subgroup Analysis Stratified by the Presence of Sarcopenia and Sex

In total, 10 of 19 female patients and 10 of 16 male patients were diagnosed with PM-defined sarcopenia, according to a previous Asian study [20]. Patients with sarcopenia were significantly older (63.2 ± 8.8 vs. 55.5 ± 7.6, *p* = 0.010), with a lower BMI (23.0 ± 2.8 vs. 25.9 ± 3.5, *p* = 0.009), creatinine level (7.9 ± 3.3 vs. 10.6 ± 4.3, *p* = 0.041), PM area (9.8 ± 4.2 vs. 16.4 ± 5.5, *p* < 0.001) and index (3.65 ± 1.50 vs. 6.00 ± 1.65, *p* < 0.001) than non-sarcopenia.

**Figure 4:**
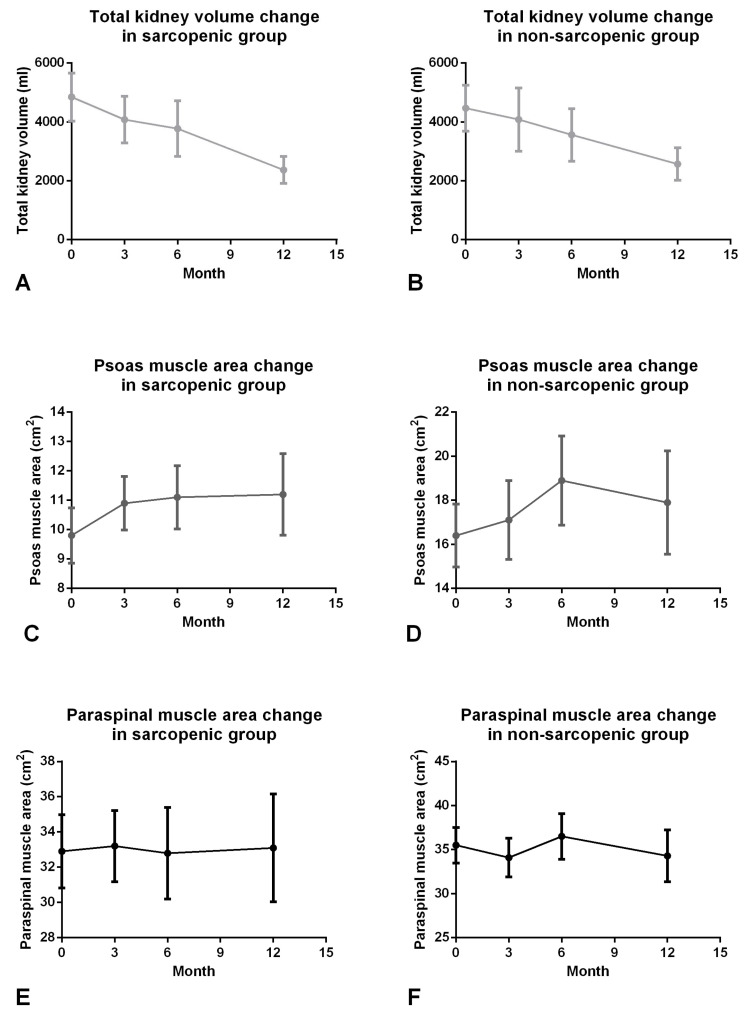
Changes in total kidney volume, psoas and paraspinal muscle areas between sarcopenic and non-sarcopenic group. A continuous reduction in total kidney volume was observed over the 12-month follow-up period in both the sarcopenic and non-sarcopenic groups (**A**,**B**). The psoas muscle showed a sustained and significant increase in the sarcopenic group, whereas it peaked at 6 months in the non-sarcopenic group (**C**,**D**). There is no significant change in paraspinal muscle area during 12-month follow-up in sarcopenic or non-sarcopenic groups (**E**,**F**).

TKV continuously decreased in 3-, 6-, and 12-month follow-up compared to baseline images in sarcopenic (4847 ± 3658 vs. 4079 ± 3456, 3772 ± 3661, 2368 ± 1664, all *p* < 0.05; Figure 4A and Table 3) and non-sarcopenic groups (4467 ± 3033 vs. 4080 ± 3719, 3562 ± 3219, 2567 ± 1828; Figure 4B and Table 3). The PM are at 3-, 6-, and 12-month follow-ups were also significantly larger than the baseline in the sarcopenic group (9.8 ± 4.2 vs. 10.9 ± 4.0, 11.1 ± 4.2, 11.2 ± 5.0, all *p* < 0.05; Figure 4C and Table 3). The PS area at 6-, and 12-month follow-up after RAE also had a significant increase compared to the baseline in the sarcopenic group (32.0 ± 9.3 vs. 32.8 ± 10.1, 33.1 ± 11.0, all *p* < 0.05; Figure 4E and Table 3) but not significant at the 3-month follow-up after RAE. On the other hand, the PM and PS only increased in the 6-month follow-up but did not differ significantly in 3- and 12-month follow-ups in the non-sarcopenic group (Figure 4D,F).

For the gender difference, TKV decreased significantly in 3-, 6-, and 12-month follow-ups compared to baseline images in female and male patients. The PM area and index continuously increased from baseline to the 12-month follow-up in male patients but became stable from the 6-month to the 12-month follow-up in female patients (Table 4).

## 4. Discussion

This study demonstrated that RAE could effectively decrease TKV and significantly increase PM and PS mass in patients with PKD. Although PM and PS mass increased after RAE, BW and BMI did not differ during 3 to 12-month follow-ups. Sarcopenia is common in patients with CKD because muscle loss is associated with aging and the progression of CKD [1,22]. Furthermore, several studies have also shown that sarcopenia is associated with mortality in patients undergoing hemodialysis or peritoneal dialysis [3,23]. Therefore, possible nutrition supplements, medication, or procedures to recover muscle loss are needed, but many clinical trials have failed to improve muscle mass [24]. Moreover, a recent study indicated that muscle mass was negatively correlated with TKV [10], and another study also demonstrated that minimally invasive intervention may increase muscle mass in patients with liver cirrhosis [25]. Transcatheter RAE is generally preferred over traditional surgery because it is a minimally invasive procedure associated with lower perioperative morbidity and mortality, particularly in patients with ESKD or multiple comorbidities. In addition, it avoids general anesthesia, allows selective targeting of the renal vasculature, and is associated with shorter recovery time compared with surgical nephrectomy [11,12,13,14,15,16]. In our cohort, no major adverse events related to the procedure were observed. Although transient post-embolization pain occurred in a subset of patients, it was self-limited and managed conservatively. No patients experienced significant hemoglobin decline or required blood transfusion following the procedure.

There are many methods to evaluate muscle mass. Previous studies have shown that cross-sectional studies are more robust and stable than bioimpedance and dual-energy X-ray absorptiometry without interference by dialysate or ascites [26,27,28]. CT-based muscle measurement also correlated with lean body mass well [23]. Furthermore, both CT and MRI might evaluate the increment of PM and PS at the same time [25]. The increment of PM and PS mass may imply different mechanisms. The PM is the only flexor connecting the hip girdle, while PS provides postural support of the lumbar spine among the abdominal skeletal muscle. Therefore, CT and MRI can clarify specific muscle groups contributing to particular functions. Many recent studies also proposed PM or PS-defined sarcopenia as a prognostic predictor in patients with pancreatic cancer, hepatocellular carcinoma, liver cirrhosis, and undergoing peritoneal dialysis [23,25,29,30,31,32].

CT and MRI are also essential for pre-operative evaluation before RAE, which can demonstrate the origin of renal arteries from the aorta and estimate TKV by the ellipsoid formula. In patients with PKD, renal parenchyma is replaced by a lot of fluid in the renal cysts. Our study revealed that TKV may be high, up to 4.5 L, and may deviate from the lean body mass based on creatinine kinetics [33,34]. The effect of shrinkage of kidney size in our study is around 50% of the original TKV after RAE, similar to previous studies in patients under hemodialysis or before renal transplantation [11,13]. We also used coils for embolization, a permanent embolizer with a long-term effect and lower side effects than ethanol [16]. Our results also indicated a persistent decrease in TKV from RAE up to 12-month follow-up. On the other hand, the PM and PS had risen to a 6-month follow-up but remained steady at a 12-month follow-up. In addition to symptom relief and muscle gain, RAE may offer an additional clinical benefit by facilitating subsequent renal transplantation. In our cohort, RAE resulted in increased space within the iliac fossa in one case, which may simplify graft kidney placement during transplantation, particularly in patients with PKD characterized by massively enlarged native kidneys. Importantly, no patients required traditional surgery during follow-up, suggesting that RAE can serve as an effective and durable alternative to surgical intervention in selected patients.

We further analyzed the subgroup data stratified by the presence of sarcopenia. We found a continuous increase in PM and PS indices in the sarcopenic group but not in the non-sarcopenic group. The possible reason may be a lower baseline of muscle mass in the sarcopenic group and a relatively higher baseline in the non-sarcopenic group, which made it easier to saturate despite the persistent decrease in TKV. Therefore, the RAE may increase muscle mass more effectively in the sarcopenic group.

In addition, we conducted a subgroup analysis stratified by sex. Although the absolute values of AC, TKV, PM area, and index were lower in females than males, the RAE reduced TKV and increased the PM similarly. Finally, we also found that the BW and BMI decreased until the 6-month follow-up but increased in the 12-month follow-up (Figure 3). The dynamic change in TKV and muscle mass was the possible reason to explain the curve change. However, the persistent decrease in AC still contributed to relieving abdominal distention. As previous studies indicated, the relief of symptoms and the restoration of muscle mass contribute to increased quality of life and lung function [13,35].

Our study has some limitations. First, it is a single-center study with a small number of patients. Nonetheless, our RAE procedure and results are similar to those of other study groups [12,14,16], so the generality to other institutes is possible. The number of enrolled patients in the entire cohort or subgroup analyses is also beyond the sample size requirement (*n* = 8) in our study design, and we use a paired comparison to minimize the sample size. Second, we did not use the volumetric method to calculate TKV or muscle mass. Although the volumetric method is more accurate for measuring TKV, processing the three-dimensional images is time-consuming. Most studies and guidelines also approved using some formula to estimate TKV [18,36,37,38,39]. Additionally, many studies have revealed a high correlation between the PM area and volume [32,33,34,35], so the PM area at the 3rd lumbar represents PM volume but is very time-saving. Therefore, further large-scale studies with automatic kidney and psoas muscle segmentation are expected. Third, CT and MRI were used interchangeably to assess TKV, PM, and PS based on clinical availability and patient compliance. Although previous studies have used CT and/or MRI to evaluate the change in TKV, PM, and PS [10,24], the concordance of quantitative measurements between CT and MRI has not been fully validated; therefore, direct comparisons between CT- and MRI-derived parameters were not performed. This may limit the comparability of muscle mass assessments across imaging modalities. Fourth, dietary management was provided according to routine clinical practice for dialysis patients; however, detailed dietary intake data were not systematically collected in this study. Finally, there was no mortality in our study during follow-up. The long-term benefits of RAE on survival have been shown [40], but PM change should be further investigated.

## 5. Conclusions

In conclusion, RAE can effectively decrease TKV and increase muscle mass in PKD patients. The increment of PM and PS was more evident in the sarcopenic group than in the non-sarcopenic group. The RAE showed significant benefits in PKD patients.

## Figures and Tables

**Table 1:** Patients’ characteristics.

Characters	Total (*n* = 35)	Sarcopenia (*n* = 20)	Non-Sarcopenia (*n* = 15)	*p*-Value	Hemodialysis (*n* = 28)	Peritoneal Dialysis (*n* = 7)	*p*-Value
Age (year)	59.9 ± 9.1	63.2 ± 8.8	55.5 ± 7.6	0.010 *	61.1 ± 8.5	54.9 ± 10.3	0.101
Female	19 (54.3%)	10 (50.0%)	9 (60.0%)	0.557	14 (50.0%)	5 (71.4%)	0.415
Body weight (kg)	64.9 ± 14.8	61.0 ± 13.5	70.3 ± 15.1	0.064	64.8 ± 15.0	65.6 ± 14.8	0.902
Body height (cm)	163.5 ± 10.0	162.9 ± 11.3	164.4 ± 8.3	0.657	164.0 ± 10.4	161.7 ± 9.0	0.602
BMI (kg/m^2^)	24.2 ± 3.4	23.0 ± 2.8	25.9 ± 3.5	0.009 *	24.0 ± 3.2	25.0 ± 4.3	0.493
AC (cm)	96.7 ± 11.0	94.9 ± 10.1	99.0 ± 12.0	0.281	97.0 ± 10.8	95.4 ± 12.6	0.746
Albumin (g/dL)	3.8 ± 0.5	3.7 ± 0.5	4.0 ± 0.4	0.076	3.9 ± 0.5	3.7 ± 0.5	0.409
Hemoglobin (g/dL)	11.0 ± 2.0	10.5 ± 2.0	11.7 ± 1.9	0.073	11.1 ± 2.1	10.4 ± 1.6	0.428
Creatinine (mg/dL)	9.0 ± 4.0	7.9 ± 3.3	10.6 ± 4.3	0.041 *	7.9 ± 3.0	13.4 ± 4.5	0.001 *
eGFR (mL/min/1.73 m^2^)	7.0 ± 5.6	7.3 ± 3.0	6.6 ± 8.0	0.719	7.9 ± 5.9	3.6 ± 1.3	0.068
Dialysis vintage (month)	70.8 ± 62.9	70.9 ± 71.7	70.6 ± 51.2	0.988	74.8 ± 68.7	54.5 ± 27.5	0.234
TKV (mL)	4684 ± 3361	4847 ± 3658	4467 ± 3033	0.746	5005 ± 3588	3403 ± 1923	0.266
PM area (cm^2^)	12.6 ± 5.8	9.8 ± 4.2	16.4 ± 5.5	<0.001 *	12.3 ± 5.8	14.1 ± 6.0	0.446
PM index (cm^2^/m^2^)	4.66 ± 1.94	3.65 ± 1.50	6.00 ± 1.65	<0.001 *	4.54 ± 1.95	5.14 ± 1.95	0.467
PS area (cm^2^)	33.5 ± 8.8	32.0 ± 9.3	35.5 ± 7.8	0.246	33.4 ± 8.9	33.7 ± 8.7	0.932
PS index (cm^2^/m^2^)	12.26 ± 2.21	11.80 ± 2.29	12.87 ± 2.03	0.162	12.18 ± 2.23	12.57 ± 2.30	0.681

They are presented as mean ± standard deviation or number (percentage). Abbreviations: BMI: body mass index; AC: abdominal circumference; eGFR: estimated glomerular filtration rate; TKV: total kidney volume; PM: psoas muscle; PS: paraspinal muscle and *: *p* < 0.05.

**Table 2:** Clinical and imaging findings change after renal artery embolization.

Characters	Baseline (*n* = 35)	3-Month Follow-Up (*n* = 31)	*p*-Value	6-Month Follow-Up (*n* = 28)	*p*-Value	12-Month Follow-Up (*n* = 24)	*p*-Value
Body weight (kg)	64.9 ± 14.8	63.2 ± 14.9	0.306	59.7 ± 13.9	0.178	61.8 ± 15.7	0.127
BMI (kg/m^2^)	24.2 ± 3.4	23.9 ± 3.4	0.327	23.3 ± 4.0	0.069	23.8 ± 3.8	0.260
AC (cm)	96.7 ± 11.0	95.3 ± 10.4	0.147	94.6 ± 12.3	0.095	93.1 ± 10.8	0.380
Albumin (g/dL)	3.8 ± 0.5	3.7 ± 0.5	0.582	3.7 ± 0.5	0.718	3.9 ± 0.6	0.433
Hemoglobin (g/dL)	11.0 ± 2.0	10.8 ± 1.6	0.490	10.6 ± 1.7	0.693	10.4 ± 1.7	0.757
Creatinine (mg/dL)	9.0 ± 4.0	9.3 ± 3.4	0.357	10.2 ± 4.2	0.366	9.1 ± 3.8	0.999
eGFR (mL/min/1.73 m^2^)	7.0 ± 5.6	7.4 ± 6.1	0.170	6.4 ± 4.0	0.719	7.7 ± 8.7	0.360
TKV (mL)	4684 ± 3361	4079 ± 3456	<0.001 *	3675 ± 3401	<0.001 *	2459 ± 1706	<0.001 *
PM area (cm^2^)	12.6 ± 5.8	13.3 ± 5.7	0.002 *	14.7 ± 6.9	<0.001 *	14.3 ± 7.1	0.001 *
PM index (cm^2^/m^2^)	4.66 ± 1.94	4.87 ± 1.80	0.023 *	5.43 ± 2.22	<0.001 *	5.33 ± 2.35	0.002 *
PS area (cm^2^)	33.5 ± 8.8	32.9 ± 8.3	0.490	34.5 ± 9.7	0.013 *	33.8 ± 10.3	0.426
PS index (cm^2^/m^2^)	12.26 ± 2.21	12.26 ± 2.07	0.745	12.79 ± 2.44	0.011 *	12.75 ± 2.71	0.179

They are presented as mean ± standard deviation. Abbreviations: BMI: body mass index; AC: abdominal circumference; eGFR: estimated glomerular filtration rate; TKV: total kidney volume; PM: psoas muscle; PS: paraspinal muscle and *: *p* < 0.05.

**Table 3:** Imaging finding changes stratified by the presence of sarcopenia.

**Sarcopenic Group**	**Baseline** **(** ***n*** ** = 20)**	**3-Month Follow-Up** **(** ***n*** ** = 19)**	***p*** **-Value**	**6-Month Follow-Up** **(** ***n*** ** = 15)**	***p*** **-Value**	**12-Month Follow-Up** **(** ***n*** ** = 13)**	***p*** **-Value**
AC (cm)	94.9 ± 10.1	94.0 ± 10.0	0.325	90.6 ± 10.6	0.104	91.5 ± 9.8	0.626
TKV (mL)	4847 ± 3658	4079 ± 3456	0.001 *	3772 ± 3661	0.004 *	2368 ± 1664	0.001 *
PM area (cm^2^)	9.8 ± 4.2	10.9 ± 4.0	0.015 *	11.1 ± 4.2	<0.001 *	11.2 ± 5.0	0.001 *
PM index (cm^2^/m^2^)	3.65 ± 1.50	4.05 ± 1.27	0.049 *	4.13 ± 1.25	<0.001 *	4.23 ± 1.83	0.008 *
PS area (cm^2^)	32.0 ± 9.3	32.2 ± 8.8	0.933	32.8 ± 10.1	0.034 *	33.1 ± 11.0	0.016 *
PS index (cm^2^/m^2^)	11.80 ± 2.29	11.95 ± 2.15	0.826	12.40 ± 2.47	0.027 *	12.85 ± 2.61	0.009 *
**Non-Sarcopenic Group**	**Baseline** **(** ***n*** ** =15)**	**3-Month Follow-Up** **(** ***n*** ** = 12)**	***p*** **-Value**	**6-Month Follow-Up** **(** ***n*** ** = 13)**	***p*** **-Value**	**12-Month Follow-Up** **(** ***n*** ** = 11)**	***p*** **-Value**
AC (cm)	99.0 ± 12.0	97.3 ± 11.1	0.310	98.9 ± 13.0	0.512	95.1 ± 12.0	0.086
TKV (mL)	4467 ± 3033	4080 ± 3719	0.051	3562 ± 3219	<0.001 *	2567 ± 1828	0.001 *
PM area (cm^2^)	16.4 ± 5.5	17.1 ± 6.2	0.059	18.9 ± 7.3	0.023 *	17.9 ± 7.8	0.205
PM index (cm^2^/m^2^)	6.00 ± 1.65	6.17 ± 1.80	0.275	6.92 ± 2.18	0.027 *	6.64 ± 2.23	0.140
PS area (cm^2^)	35.5 ± 7.8	34.1 ± 7.6	0.407	36.5 ± 9.3	0.114	34.3 ± 9.8	0.259
PS index (cm^2^/m^2^)	12.87 ± 2.03	12.75 ± 1.91	0.820	13.23 ± 2.42	0.136	12.64 ± 2.94	0.574

They are presented as mean ± standard deviation. Abbreviations: AC: abdominal circumference; TKV: total kidney volume; PM: psoas muscle; PS: paraspinal muscle and *: *p* < 0.05.

**Table 4:** Imaging finding changes stratified by sex.

**Female**	**Baseline** **(** ***n*** ** = 19)**	**3-Month Follow-Up** **(** ***n*** ** = 18)**	***p*** **-Value**	**6-Month Follow-Up** **(** ***n*** ** = 17)**	***p*** **-Value**	**12-Month Follow-Up** **(** ***n*** ** = 16)**	***p*** **-Value**
AC (cm)	89.7 ± 7.2	89.1 ± 7.5	0.731	88.1 ± 8.8	0.369	87.3 ± 7.6	0.265
TKV (mL)	3202 ± 1635	2656 ± 1449	0.001 *	2244 ± 1220	<0.001 *	1916 ± 1197	<0.001 *
PM area (cm^2^)	9.5 ± 3.7	10.3 ± 3.3	0.004 *	10.9 ± 3.6	0.002 *	10.6 ± 3.2	0.024 *
PM index (cm^2^/m^2^)	3.79 ± 1.44	4.11 ± 1.18	0.055	4.35 ± 1.27	0.015 *	4.31 ± 1.35	0.034 *
PS area (cm^2^)	28.6 ± 6.2	27.8 ± 4.4	0.640	29.4 ± 6.5	0.098	29.0 ± 6.5	0.509
PS index (cm^2^/m^2^)	11.47 ± 2.14	11.39 ± 1.65	0.816	11.88 ± 2.23	0.056	11.88 ± 2.45	0.263
**Male**	**Baseline** **(** ***n*** ** =16)**	**3-Month Follow-Up** **(** ***n*** ** = 13)**	***p*** **-Value**	**6-Month Follow-Up** **(** ***n*** ** = 11)**	***p*** **-Value**	**12-Month Follow-Up** **(** ***n*** ** = 8)**	***p*** **-Value**
AC (cm)	104.9 ± 8.8	103.9 ± 7.2	0.106	104.0 ± 10.7	0.104	104.8 ± 5.1	0.958
TKV (mL)	6445 ± 4039	6048 ± 4433	0.016 *	5886 ± 4471	0.007 *	3545 ± 2113	0.007 *
PM area (cm^2^)	16.4 ± 5.6	17.5 ± 5.8	0.077	20.6 ± 6.8	0.005 *	21.6 ± 7.2	0.016 *
PM index (cm^2^/m^2^)	5.69 ± 1.99	5.92 ± 2.02	0.219	7.09 ± 2.39	0.008 *	7.38 ± 2.67	0.038 *
PS area (cm^2^)	39.3 ± 7.9	39.9 ± 7.3	0.629	42.6 ± 8.4	0.070	43.3 ± 10.0	0.682
PS index (cm^2^/m^2^)	13.19 ± 1.97	13.46 ± 2.03	0.829	14.18 ± 2.14	0.111	14.50 ± 2.45	0.504

They are presented as mean ± standard deviation. Abbreviations: AC: abdominal circumference; TKV: total kidney volume; PM: psoas muscle; PS: paraspinal muscle and *: *p* < 0.05.

## Data Availability

The data underlying this study are not publicly available due to privacy and ethical restrictions. The data may be available from the corresponding author upon reasonable request and with approval from the institutional review board.

## Abbreviations

The following abbreviations are used in this manuscript:

CKD	Chronic kidney disease
ESRD	End-stage renal disease
PKD	Polycystic kidney disease
TKV	Total kidney volume
RAE	Renal artery embolization
PM	Psoas muscle
PS	Paraspinal muscle
L3	The third lumbar vertebral
BH	Body height
NTUH	National Taiwan University Hospital
CT	Computed tomography
MRI	Magnetic resonance imaging
BW	Body weight
BMI	Body mass index
AC	Abdominal circumference
eGFR	Estimated glomerular filtration rate
TR	Repetition time
TE	Time to echo
HASTE	Half-Fourier acquisition single-shot turbo spin-echo
L	Longitudinal length
W	Maximal width
D	Maximal depth
